# Paving a pathway for large-scale utilization of genomics in precision medicine and population health

**DOI:** 10.3389/fsoc.2023.1122488

**Published:** 2023-05-19

**Authors:** Nephi A. Walton, G. Bryce Christensen

**Affiliations:** Intermountain Precision Genomics, Intermountain Healthcare, Salt Lake City, UT, United States

**Keywords:** precision medicine, genomics, population health genomics, bioinformatics, whole genome sequencing, implementation science, genetic testing, reimbursement

## Abstract

Having worked with two large population sequencing initiatives, the separation between the potential for genomics in precision medicine and the current reality have become clear. To realize this potential requires workflows, policies, and technical architectures that are foreign to most healthcare systems. Many historical processes and regulatory barriers currently impede our progress. The future of precision medicine includes genomic data being widely available at the point of care with systems in place to manage its efficient utilization. To achieve such vision requires substantial changes in billing, reimbursement, and reporting as well as the development of new systemic and technical architectures within the healthcare system. Clinical geneticist roles will evolve into managing precision health frameworks and genetic counselors will serve crucial roles in both leading and supporting precision medicine through the implementation and maintenance of precision medicine architectures. Our current path has many obstacles that hold us back, leaving preventable deaths in the wake. Reengineering our healthcare systems to support genomics can have a major impact on patient outcomes and allow us to realize the long-sought promises of precision medicine.

## Introduction

The separation between the potential for precision medicine and the current reality have become abundantly clear. This became evident in my work with two large population sequencing initiatives, the MyCode program at Geisinger (Carey et al., 2016), and the HerediGene program at Intermountain Healthcare (IH) (Walton et al., 2022). As a clinical geneticist with a professional background in programming and information technology, I have gained insight into both the clinical application of genomics as well as the technical infrastructures required to support it. At both Geisinger and IH, hundreds of thousands of patients have available sequencing data, creating a daily struggle to push actionable data into the clinical space across a large healthcare system. To do so efficiently and at scale requires workflows, policies, and technical architectures that are foreign to most healthcare systems (Walton et al., 2022). Many historical processes and regulatory barriers currently impede the ability to realize the ultimate vision of precision medicine (Klein, 2020; Walton et al., 2020, 2021; Abdelhalim et al., 2022; Schaibley et al., 2022; Stenzinger et al., 2022). Technology and the cost of sequencing are no longer significant impediments in the US healthcare system, but rather the efficient and proper utilization of such technologies holds us back. New possibilities depend on our ability to diverge from conventional processes justified primarily by historical context rather than current utility. While some of these barriers are necessary to ensure patient safety and allow for evidence-based approaches, there is certainly ample room for improvement and failure to increase efficiencies may leave us well behind. Many historical conventions lose relevance in the face of new technologies and scientific discovery. While other industries blossom through the grasp of these technologies, medicine grinds slowly forward. Additionally, newly proposed regulatory measures may also threaten innovation and progress in this space (HR4128, 2021; ACMG Group Sign-on Letter, 2022; FDA, 2022; HER, 2022).

Sequencing, aside from interpretation, is rapidly approaching the cost of other regularly ordered laboratory tests, with the $100 genome clearly within our grasp (Philippidis, 2022). Several initiatives exist that use whole genome sequencing (WGS) for newborn screening (Buxton, 2022), and it is likely that in the future, genome sequence will be part of every medical record. Healthcare systems must be prepared for the tsunami of clinically actionable information generated from this data. We have previously described IH's work to deploy a precision health framework (Walton et al., 2022). As we pushed through this deployment, glaring deficiencies presented themselves as barriers to fully realizing precision medicine. This perspective presents a vision for precision medicine with suggestions to expedite that vision into daily clinical care. This vision is based on my experience with genomics in the US healthcare system, though, some of these challenges may be encountered in other healthcare systems around the world.

## The vision

Genetic testing would no longer consist of a thousand different orderable panels, as is the case today, but would instead consist of one genetic test for all purposes that could be used throughout the life of the patient. Any need for genetic interrogation would automatically initiate the process of WGS using testing modalities that capture the spectrum of genomic variation, including structural changes. The data from this test would be readily available at the point of care for a myriad of purposes, including but not limited to:

- Pharmacogenomics—Prescribing medications according to an individual's genetic profile to deliver the most suitable medication and dosage for optimal results based on their genetic characteristics.
- Gene based therapeutics—Identification of patients with disease that have genetic variation that is responsive to gene therapies or biologics and initiating those treatments.
- Disease prevention—Identification and implementation of preventative action on individuals who harbor pathogenic variants in genes known to predispose to preventable disease, including, but not limited to, those recommended for reporting by the American College of Medical Genetics (ACMG) (Miller et al., 2021).
- Disease risk modeling—Deployment of scoring models to predict patient disease and enable prevention. Such models would include polygenic risk scores and more complex models that incorporate genomic information with other patient data and/or environmental information to predict and prevent disease.
- Real-time genetic diagnosis—Facilitating genetic diagnosis in real-time as patients seek medical attention at hospitals or clinics when exhibiting symptoms. Enabling the use of faster and more effective treatment options as a result of more accurate diagnoses.
- Population health genomics—The utilization of genomic data by health systems to cater disease management strategies to the genetic diseases of the population being served.
- Reproductive decision making—The use of genomic information from individuals considering having children to help them understand carrier risks and make informed reproductive decisions that may include the use of in vitro fertilization and preimplantation genetic diagnosis.

This genomic information would travel with the patient to different hospitals and clinics in different states or countries. The clinical use of this data would be achieved almost entirely through approved bioinformatics systems that are not subject to manual review and medical director sign-out, enabling the inexpensive and rapid utilization of genomic data. Exceptions would be made for diagnostic cases that involve variation or genes that are not well characterized.

Complex clinical decision support systems (CDSS) use the genomic data to augment physician judgement, guiding them through genomic specific care pathways, adjusting prescriptions according to a patient's predicted response to medications and automatically scheduling preventative maintenance and disease surveillance. Similar systems are deployed through mobile and wearable devices that monitor and guide patient health outside the clinical setting, enabling patients to actively participate in their care. Complex artificial intelligence (AI) models using genomics project patient trajectory and allow for adjustment so the patient can reach their optimal health targets.

Clinical geneticists begin serving more in administrative roles overseeing the application of genomics and precision medicine across healthcare systems, with titles such as Chief Genomics Officer (CGO) or Chief Precision Medicine Officer (CPMO). Such officers would work with each department in the system to apply precision medicine technologies and practices to their respective domains. Clinical geneticists would retain limited practice in diagnosis and management of rare disorders, with many genetic conditions distributed to other specialties. Genetic counselors would become nearly as ubiquitous as nurses to facilitate the deployment and management of precision health systems.

## The change

To fully scale and implement the aforementioned vision requires rethinking and re-engineering many processes that have been in place for decades. Though not exhaustive, the list below addresses some critical changes needed to achieve a more complete realization of precision medicine.

### New paradigm for ordering and billing genetic testing

Currently, billing for sequencing is messy. Labs have found creative ways to bill that meet payers' often ill-informed demands. As an example, some payers require single gene tests before sending a panel, and others might require a panel before an exome. Today, all these tests are essentially run on the same platform, so billing for what is allowed by the payer and then reflexing to obtain more analysis such as a panel, extended panel, exome, or genome is not uncommon. This results in laboratories, who really only offer one product (genome or exome sequencing), lessening the product's value and complicating the diagnostic process to meet the demands of payers. All that comes back from these tests, which are often run on an exome or genome backbone, is a PDF that displays maybe a handful of variants, leaving gigabytes of clinically useful information outside the healthcare system.

Genetic testing should follow a model similar to medical imaging. Genome sequencing should no longer be billed as a laboratory test. It should be billed as a procedure like an MRI. This “procedure” is ordered the first time there is any indication for any genetic testing and generates sequence data accessible to the healthcare system. The interpretation occurs thereafter and would be performed and billed separately, like a radiologist billing for reading an MRI. The complete data from the genome sequencing would remain accessible to the healthcare system for serial interrogation based on the longitudinal needs of the patient. Also like an MRI, specialist physicians should be able to browse this data with the aid of bioinformatics tools at the point of care in the context of the patient. If they find something that impacts patient care, they should be able to request that this finding be reassessed by the interpretation service. Interpretation should be able to occur onsite with the aid of bioinformatic tools or remotely using specialized services. Currently, most of the cost of clinical genome sequencing is in the interpretation, not in the laboratory process. This change would make genome sequencing a readily available and inexpensive commodity and facilitates less expensive bioinformatic interpretation where there is less ambiguity around variant consequences. It would also open up substantial opportunity for research that can make the genomic data even more useful and its interpretation cheaper.

### Automated bioinformatic analysis and reporting

Manual sign-out of genetic testing reports can be automated in areas where genomic variation is well understood, and laboratory processes are well defined with high levels of accuracy. Clinical Laboratory Improvement Amendments (CLIA) regulation is administered by Centers for Medicare & Medicaid Services (CMS) to ensure laboratory testing is performed accurately, reliably, and with consistent quality. Sequencing data meeting certain quality metrics coming from a CLIA certified laboratory should be able to be processed entirely by approved bioinformatics processes. These processes could provide results were there is certainty around variant classification, such as with pharmacogenomics, polygenic risk scores, and ClinVar variants with high levels of evidence. ClinVar is a publicly available database of genetic variations and their clinical significance, that is commonly used to interpret genetic test results. ClinVar variants with a 3-star designation have been reviewed by an expert panel and achieved consensus in classification and 4-star variants are considered a practice guideline. Variants with a 3-star or 4-star designation should be able to be reported in an automated fashion. Many laboratories have already automated much of this reporting while still maintaining manual signoff for the report.

As the majority of variant evaluation and sign out is clinically assessed through the analysis of bioinformatically generated information, it makes sense that these processes can be automated by analyzing the human processes taking place and replacing them with computer algorithms so long as they reach a certain threshold of validity. Arguments against trusting a computer for such processes fail in that all the information used to sign out such reports is generated by a computer, there is no human sense such as vision that is used in traditional pathology that gives a human an advantage over a machine when reporting well established variants. For example, in my experience as a laboratory medical director signing out pharmacogenomic testing reports, we had a standard pipeline and protocols, reporting only well-known variants that do not need evaluation every time they are seen. Deviations from this standard process in pharmacogenomics reporting is rare. There is no more need for a human in this process then there is for reporting a complete blood count (CBC). Of course, there will be exceptions that require medical director evaluation when things do not meet certain specifications, as is the case with any laboratory test. Additionally, there would be a medical director responsible for oversight and sign off on the process, but there would be no need evaluate each individual's report. As our knowledge about genetic variation increases, the majority of this information will be able to be reported without human intervention. While automated sign out of reports is certainly not standard practice in the field of pathology, it is something that needs to take place as the volume and use of genomic data grows and the capabilities of artificial intelligence improve and even begin to exceed that of humans. These automated reporting systems sitting on top of large amounts of genomic data can serve as powerful tools to further precision medicine and its translation to patient care. Such systems would allow for automated reanalysis and reporting on existing genomic data as new information about genomic variation becomes available. The implementation of these technical architectures would enable inexpensive reporting/interpretation on already inexpensive genome sequencing, providing significant value for genome sequencing at a very low cost.

### Dynamic electronic genetic test reports

It is not uncommon for knowledge about genetic variation to change over time. This can be problematic when a change requires the reissuance of thousands of clinical reports and notifying equal numbers of clinicians and patients of the change. The dynamic nature of genetic information lends itself to dynamic electronic reporting that keeps up with the current science and automates updates to clinicians and patients. This type of reporting may also be our solution for variants of unknown significance (VUS), which currently pose a significant clinical problem (Richter, 2013). Utilizing dynamic electronic reporting, it is possible to restrict the viewing of variants whose clinical utility is uncertain to specialist providers. Such physicians may change the interpretation of the variant and push it to the patient report if they feel confident that the variant is impactful. This would avoid confusing and inducing anxiety in patients while preventing providers with little genetic experience from misinterpreting and misusing information from the final report. We need to build a dynamic reporting system that responds appropriately to new information.

### Genome first approach

Historically, the **first** visit with a geneticist involved seeing the patient and performing a very detailed physical exam to identify the right genetic test to order. As we move to genome sequencing as the first-line test, the value of this **first** visit and exhaustive phenotyping become questionable. We have begun this transition at IH, the challenge being that many payers will not pay for genetic testing unless the patient has seen a clinical geneticist. Despite this challenge, we have reduced our **first** visit encounters by 50% by implementing it where payers have allowed. Furthermore, many patients do not need to see a clinical geneticist at all with this approach because their resulting genetic conditions are referred to the specialist, who ultimately treats that condition with a genetic counselor providing counseling around inheritance and disease risk. In our early experience with this approach, it is far more valuable to have the genetic test available when seeing the patient and tailoring the exam and questioning to the discovered genetic variation. It is still required to have a major phenotype to base the initial genomic analysis on, which can often be garnered from the referral. The sequencing **first** approach becomes especially important with the current shortage of clinical geneticists and long wait times (Dragojlovic et al., 2020; Simon et al., 2022). Today, phenotype still holds significant value as we try to tease out the clinical implications of variation in 20,000 genes, but the diagnostic utility of exhaustive phenotyping is waning, and the sequencing **first** approach is gaining ground. Working with payers to clearly designate phenotype algorithms in each specialty for which genetic testing can be ordered without a prior visit to a geneticist can help drive this forward. This would ultimately save payers money by eliminating an unnecessary encounter. A brief genetic counseling session is still required to initiate the testing but even this part could arguably be replaced with technology over time.

### Enabling first line providers to order genetic testing

As the importance of detailed phenotyping lessens, opportunity arises for other, less specialized providers to initiate the genome sequencing process. Pediatricians, for example, are qualified to diagnose intellectual disability (ID). The current first-line testing modality for ID includes WGS. We can enable this process with informatics support to manage the pre-counseling aspects of the test and population health genomics architectures to manage the secondary findings (Walton et al., 2022). Epilepsy also includes WGS as a first line test (Smith et al., 2022). It does not make sense to have a clinical geneticist in the care pathway for epilepsy unless the diagnosis is a complex syndrome affecting other systems since the patient will ultimately be managed by neurologists anyway. Shifting the ordering of genetic testing from clinical genetics to other providers increases the throughput of the system, thereby increasing the number of genetic tests ordered and resulting diagnoses received. We have already seen this taking place in neurology departments where there is a movement to have dedicated neurology genetic counselors who facilitate this workflow (Wofford et al., 2019). One key factor to enabling this approach is to have an infrastructure that allows for routing results to a clinical geneticist for support when necessary. Improving genomics education of frontline providers is also important. The amount of education required would not be extensive and could easily be integrated into both medical school and residency training programs.

### Role of genetic counselors and genetic counseling assistants

As we enable primary care and other specialties outside of clinical genetics to take on genetic testing, genetic counselors are still critical to making these systems work. With our public health genomics deployment, we were quick to realize the limited availability of clinical geneticists. Genetic counselors are critical to the deployment and maintenance of precision medicine at scale. Furthermore, providing genetic counselor assistants to support genetic counselors increases their availability and productivity (Krutish, 2022), with the added benefit of augmenting the genetic counseling pipeline with high-quality applicants with significant field exposure. While informatics frameworks can help deliver a great deal of precision medicine, the field requires trained individuals to deploy, maintain, and operate complex frameworks. Genetic counselors have proven to have a very robust set of skills, performing well in diverse roles that support such frameworks. Their base set of skills will need to be expanded with genetic counselor training programs including more exposure to polygenic risk scores (PRS) and their associated relationships to complex disease as well as an increased exposure to pharmacogenomics. While oversight of complex precision medicine frameworks by a clinical geneticist may be desirable, such systems cannot be too dependent on this scarce resource. Increasing the number of genetic counselors in the system is critical to the success of any precision medicine program.

### Increased clinical use of sequencing leads to increased clinical utility

Clinical genetics is an interesting specialty in that it sits at the intersection of clinical care and research. New discoveries are frequent but are often made through thorough clinical investigation with the intent of diagnosing and treating the patient rather than using controlled studies with established IRB protocols. Many of the genetic disorders we know about today are the result of the clinical uptake of exome sequencing. Over 500 new genetic disorders have been discovered by providers matching their clinical findings to other providers through the Matchmaker Exchange (Boycott, 2022). Without the widespread adoption of whole exome sequencing, this progress would have been impossible. Likewise, we need large cohorts of sequenced patients that provide us with the statistical power to study treatment outcomes relative to the causal gene. We currently face the challenge of increasing the clinical use and utility of WGS. The extra information provided by WGS has little utility if we do not understand its clinical implications. We learn about the clinical implications of this information through increased use of clinical WGS. This challenge is compounded with the advent of long read sequencing, which provides even more information. It is critical that we enable the rapid clinical uptake of these technologies.

### Challenging the academic model

The discovery of new variants that cause disease in a well-described gene does not get much academic attention. In fact, some of my colleagues have claimed that it's difficult to get funding for large-scale functional studies because they have lost their novelty. With the inability to publish such findings, there is little incentive to increase public knowledge on gene variation. Laboratories make significant contributions to ClinVar, but clinicians rarely do, despite being most qualified to link patient phenotype to genetic variation and ultimately determine the pathogenicity. Less than 10% of ClinVar submissions are from clinicians at the time of writing (ClinVar, 2023). Even if journals accepted publication of specific variants, the process of submitting and publishing is very inefficient, especially for clinicians whose primary focus is patient care (Vines, 2015). New academic models need to be developed that incentivize clinician contribution to public knowledge and provide an easy-to-use framework to do so. Frameworks have been proposed and even implemented with specific genes and conditions (Majumder et al., 2021). We should continue to build and improve these frameworks scaling them to all genetic conditions. As they are developed, they should account for clinician incentives, patient privacy protections, and institutional review board (IRB) requirements, to lower barriers of publication. Most clinicians will not go to the length of writing an IRB protocol to submit such findings.

### Technical architectures needed

Prior work has uncovered the deficiencies of technical workflows to facilitate precision health, especially at the interface of the laboratory and the clinic (Walton et al., 2020, 2021, 2022). These processes are critical to the success of scaling precision medicine. To my knowledge, such complete systems do not exist in the commercial space and required our organization to build custom solutions. Electronic Medical Record (EMR) vendors have begun to build infrastructure to support precision medicine (Walton et al., 2020) and third-party tools with that cover different aspects of precision medicine are beginning to appear. There remains significant work to be done in this area particularly in the domain of implementation science (Wiley et al., 2022).

### Prioritization and governance of technical architectures

In my experience advising other healthcare systems, implementation of precision medicine technical frameworks tends to be a low priority to organizational leadership. This is particularly true when projects require resources from the EMR technical team. As genomic information becomes more available and critical to daily patient care, healthcare systems that have avoided implementing such architectures will find themselves struggling to manage the data and the resultant clinical implications. This can result in suboptimal patient care and even legal liability. In my prior experience, one major barrier to achieving approval and prioritization from leadership is their concern over who will manage the domain specific aspects of the such architectures (Walton et al., 2020, 2021, 2022). Having a domain expert or CGO who can oversee and manage the deployment and continued use of such technical architectures is critical. Clinical geneticists are already filling such roles in healthcare systems that have progressed in this space but without formal recognition or title.

### Reimbursement challenges

Genetic testing and clinical genetics encounters have been poorly reimbursed (Raspa, 2021), with many clinical genetics departments operating at substantial losses. Becoming a clinical geneticist requires two additional years of training after a primary specialty, yet financial compensation is usually less than practicing in the prerequisite field. This has led to a cohort of individuals being primarily driven by scientific interest and desire to help patients with compensation as an afterthought. Having such an altruistic workforce is beneficial, but the historical lack of financial focus of the profession may be what has led to unsustainable reimbursement models and a small workforce. As the need for such services becomes more critical, there must be financial incentives to develop the clinical workforce and required infrastructures within the healthcare systems.

Perhaps one of the most important roles for genome sequencing is in preventative care. Genetic information allows for surveillance and prevention that can significantly lower morbidity and mortality for patients which ultimately decreases their long-term cost of care. While several well studied genes have demonstrated financial value (Wordsworth et al., 2010; Lázaro, 2017; Tuffaha et al., 2018), it is especially true when the impact of multiple actionable genes is considered in concert. The challenge is that these financial incentives are long term savings and there may even be short term cost increases due to the associated preventative care. United States (US) payers have not shown much interest in cost savings that cannot be realized in time periods shorter than their average churn, whereas national healthcare systems are more likely to see these financial benefits and adopt related policies. As there is a net national benefit in terms of reduction of cost, morbidity, and mortality, government intervention may be prudent to move the US forward.

Payers largely cover preventative care with grade A or B recommendations from the United States Preventative Task Force (USPTF) guidelines (HR3590, 2022). These guidelines are very conservative and slow to develop. Of the 73 American College of Medical Genetics (ACMG) actionable genes (Miller et al., 2021), only two currently have USPTF guidelines (US, 2019) despite significant evidence for the clinical impact of other genes on the list, including those for Lynch Syndrome [Evaluation of Genomic Applications in Practice Prevention (EGAPP) Working Group, 2009] and Familial Hypercholesterolemia (Lázaro, 2017). Using this slow gene-by-gene approach will take decades to realize the potential of genomic preventative care. IH currently considers over 200 genes actionable (Walton et al., 2022), as does ClinGen (ClinGen Curated Genes, 2022). The USPTF takes a very deliberate disease specific approach to evaluation. As preventative WGS and its interpretation drop in price dramatically its utility should be assessed as a whole for preventative care, rather than assessing the impact of individual genes. In most cases, the cost of testing one gene vs. 1,000 different genes is not significantly different as laboratories typically use an exome or genome backbone for testing. Therefore, opting for a more focused approach of testing a single gene does not appear practical as there is considerable added value to be obtained from the extra genomic data. This is especially true as we realize the growing list of genes that contribute to each disorder and how difficult, if not impossible, it is to differentiate between causal genes through clinical evaluation. Interestingly, an argument that has been made to justify testing only two genes rather than a panel is that primary care physicians (PCPs) may not have the expertise and time to handle the management of BRCA1 and BRCA2 let alone those related to other genes (Rajagopal et al., 2019). In my experience, few primary care physicians are prepared to manage any of these conditions if they do not have prior experience with them, and many have expressed concern about getting results from our population health sequencing programs. However, those concerns were largely allayed when the genetic testing results were delivered with clear concise management guidelines, or the patients were initiated on care pathways that were independent of their primary care provider. Rather than limiting the number of genes tested or returned to patients our focus should be on implementing infrastructures to manage this information and guide patients and providers through care pathways. This challenge is not going to get easier to tackle, it is going to grow every year as our genomic knowledge increases.

### Data storage and access

Where genomic data should flow after it is generated is an important question. As patients navigate through various health insurance plans, seek specialized medical services, and travel to different geographic locations, it is crucial for the data to be accessible to multiple interpretive services and healthcare systems. There are still open questions of what data to store (genomic variant call format (gVCF), compressed reference-oriented alignment map (CRAM)), where to store it (onsite, federated systems, central repository, flash drive), and who pays for the storage (government, patient, healthcare system, laboratory). Additionally, as the price of sequencing comes down there is a question as to whether it is cheaper to store the data or just re-sequence the patient as needed. The relative cost of data storage is growing as a contributor to the overall cost of genome sequencing. AWS introduced S3 cloud storage in 2006 at a price of $0.15 per GB per month. Standard S3 storage in 2022 is about $0.022/GB/month (depending on usage volume), nearly a seven-fold reduction in price. The cost of genome sequencing dropped by a factor of more than 20,000 during the same period, from over $20M in 2006 to less than $1000 in 2022. The 10-year cost of storing a genome for ongoing analysis on AWS has been estimated at over $300. The cost may be mitigated by use of archival storage systems or advanced data compression. **One** strategy would be to keep variant data as gVCF files in high-availability storage while storing read data in a lossless CRAM format in deep archive storage where retrieval is delayed. Over 10 years, we estimate this approach would cost about $40, allowing for the CRAM to be retrieved from the archive at least twice. Limitations to using gVCF are having an incomplete human reference genome and persisting challenges around calling of structural variants and repeat expansions, which at times necessitates the use of CRAM format for reevaluation. Strategies for how to store and access data need more definition and study to ensure seamless precision care across systems.

### Regulation

While some regulatory oversight is needed as precision medicine gains ground in the healthcare system, over-regulation could hinder progress in the field. Although often started with the intent of protecting patients, regulation can also have the effect of benefitting industry giants by creating significant barriers to market entry and thereby eliminating smaller innovative players from the market. This ultimately limits innovation by reducing competition. The recent introduction of the VALID Act (HR4128, 2021) proposing FDA regulation of genetic testing could have had a large negative impact on genomic innovation if it had passed. Such regulation would impose significant regulatory burdens and associated costs that are prohibitive to small academic laboratories who have driven a significant amount of the innovation in this field (ACMG Group Sign-on Letter, 2022). Careful analysis of actual harms vs. benefits of new regulation should be considered before any laws are passed. It is also important to realize that genetic laboratories already have oversight by other regulatory bodies. FDA regulation could impose significant braking on an industry whose rapid progress has been very beneficial to patients. With the failure of the VALID act to pass, it is uncertain what steps the FDA will take next toward regulation of genomic testing. As the FDA has asserted itself into this space, it has a responsibility to ensure that their policies enable small innovative companies to enter and operate in the market. They also need to ensure that they have the capacity to manage oversight in a way that does not impose significant financial and temporal burdens on highly innovative laboratories. The FDA's regulation of CDSS and artificial intelligence, are equally as concerning as these regulatory measures will also have profound impacts on the delivery of precision medicine (FDA, 2022; HER, 2022). Regulation is necessary and can offer protections to patients but must be employed prudently to ensure that it does not ultimately harm patients by preventing highly beneficial products from coming to market.

## Conclusion

While the world changes around us, healthcare cannot afford to stand still. Reengineering different aspects of our healthcare system to harness the power of genomics could expedite our path to reaching the full potential of precision medicine. We find ourselves at a critical intersection where we can opt to take the path of least resistance, leaving preventable deaths in our wake, or take the path of change, reducing mortality and morbidity while improving quality of life. We should implement meaningful changes to accelerate this field, as it has so much potential to impact patients and their care. This vision reflects my opinion, shaped by my experience working in two of the largest population health sequencing programs in the U.S. healthcare system. Certain principles may not be applicable to other healthcare systems, and I acknowledge that other professionals working in this field may hold divergent perspectives. My objective is to facilitate a dialogue that encourages a variety of perspectives and fosters collaboration toward leveraging genomics to advance the field of precision medicine at an accelerated pace.

## Data availability statement

The original contributions presented in the study are included in the article/supplementary material, further inquiries can be directed to the corresponding author.

## Author contributions

NW and GC performed analysis of data storage and sequencing costs for this work and contributed to the “Data Access and Storage” section of this work. Both authors contributed to the article and approved the submitted version.
